# LAILAPS-QSM: A RESTful API and JAVA library for semantic query suggestions

**DOI:** 10.1371/journal.pcbi.1006058

**Published:** 2018-03-12

**Authors:** Jinbo Chen, Uwe Scholz, Ruonan Zhou, Matthias Lange

**Affiliations:** Research Group Bioinformatics and Information Technology, Leibniz Institute of Plant Genetics and Crop Plant Research (IPK) Gatersleben, Seeland OT Gatersleben, Germany; Universite de Montreal, CANADA

## Abstract

In order to access and filter content of life-science databases, full text search is a widely applied query interface. But its high flexibility and intuitiveness is paid for with potentially imprecise and incomplete query results. To reduce this drawback, query assistance systems suggest those combinations of keywords with the highest potential to match most of the relevant data records. Widespread approaches are syntactic query corrections that avoid misspelling and support expansion of words by suffixes and prefixes. Synonym expansion approaches apply thesauri, ontologies, and query logs. All need laborious curation and maintenance. Furthermore, access to query logs is in general restricted. Approaches that infer related queries by their query profile like research field, geographic location, co-authorship, affiliation etc. require user’s registration and its public accessibility that contradict privacy concerns. To overcome these drawbacks, we implemented LAILAPS-QSM, a machine learning approach that reconstruct possible linguistic contexts of a given keyword query. The context is referred from the text records that are stored in the databases that are going to be queried or extracted for a general purpose query suggestion from PubMed abstracts and UniProt data. The supplied tool suite enables the pre-processing of these text records and the further computation of customized distributed word vectors. The latter are used to suggest alternative keyword queries. An evaluated of the query suggestion quality was done for plant science use cases. Locally present experts enable a cost-efficient quality assessment in the categories trait, biological entity, taxonomy, affiliation, and metabolic function which has been performed using ontology term similarities. LAILAPS-QSM mean information content similarity for 15 representative queries is 0.70, whereas 34% have a score above 0.80. In comparison, the information content similarity for human expert made query suggestions is 0.90. The software is either available as tool set to build and train dedicated query suggestion services or as already trained general purpose RESTful web service. The service uses open interfaces to be seamless embeddable into database frontends. The JAVA implementation uses highly optimized data structures and streamlined code to provide fast and scalable response for web service calls. The source code of LAILAPS-QSM is available under GNU General Public License version 2 in Bitbucket GIT repository: https://bitbucket.org/ipk_bit_team/bioescorte-suggestion

This is a *PLOS Computational Biology* Software paper.

## Introduction

In order to retrieve and explore database content, query interfaces are required. These are, at a simplistic view, brokers between the user’s information needs and the database content that is accessible using declarative query languages like SQL or imperative application programming interfaces (API). Human computer interfaces (HCI) make use of these APIs to provide frontends to interact with the databases.

The annual NAR database issue listed for 2017 more than 1,900 databases that are in majority accessible by individual web frontends [1]. This offered frontends ranges from closed data navigation to open, text query based search engines. Navigation and forms are focused to query facts. Query languages and keyword queries features information retrieval (IR). Factual queries retrieval answers that match precisely, while an information retrieval query attempts to find documents containing information that is relevant to an area of inquiry.

Both types of queries have pros and cons. Factual query HCIs are well optimizable, the frontend is stable, and robust. This is paid by less query flexibility and commonly complex frontends. The feature of information retrieval queries to formulate vague queries to find relevant facts is paid with a high computational effort and a higher risk to come up within complete or less relevant results. This is known as recall and precision trade-off, which is a crucial quality metrics for IR systems and an objective of a dedicated research field [2–4].

The approach to execute keyword based queries is to create an inverse text index for a particular database. This index maps a word or phrases to database records, elements, attributes etc. More abstract, this is a mapping of tokens to documents. This mapping function can be quite complex to support similarity, word steam, and phonetic matching. Furthermore, keywords need to be tokenized, i.e. decompose phrases into words, remove prefixes and punctuation, filter abundant tokens, correct spelling or map to representative vocabulary and synonyms. In general, those tasks are well parameterizable to match the particular properties of database and search engines.

In contrast, phrasing queries for genes, traits as sequences of keywords [5] to express the users search intention is a big challenge. The user has to narrow down complex facts as those keywords that most likely discriminate relevant from non-relevant database records. And this is done without knowing the terminology used in the particular database. Spelling correction and syntactic query expansion are robust and widely adopted solutions [4], but replacing possible unspecific keywords by semantic equivalent, which are more sensitive, is quite more challenging [5].

Here, an original query must be reformulated as a sequence of terms that is expected to (i) express best the users intention and (ii) match the most relevant database records [6]. In other words, query suggestion, or query expansion aims to increase the search space to reduce the false negative and keep the number of false positive documents low.

For semi-automatic query suggestion, the system offers alternative terms to the user. It is necessary to present the terms to the user in some reasonable order, preferably one in which the terms most likely to be useful are near the top. A recent survey [7] provides a useful overview to query suggestion methods and listed three important sources as basis for semantic query suggestions: (i) query logs, (ii) controlled vocabulary, and (iii) search corpus.

Log files are very valuable resources from real users’ information to generate query suggestions [8]. However, log files are privacy protected and very hard to get from public sources in a reasonable size and constantly updated. Controlled vocabulary, i.e. ontologies perform well for query suggestion [9], but have a corpus bias requiring significant expertise and curation. Corpus-based measures of word semantic similarity try to identify the degree of similarity between words using information exclusively derived from the text corpus. The drawback is the high computational costs and there is a high potential for non-relevant and misleading query suggestions [10].

The advantage of corpus-based query suggestion is to enable query suggestions without query logs and a laborious curation of controlled vocabulary. The challenge was to overcome the drawback of computational and memory complexity. After studying the literature, we decided to implement a machine learning approach called Word2Vec [11].

In subsequent sections, we will give a basic introduction to the Word2Vec algorithm, discuss details of its implementation and the training of the neural network. Finally, a test set of representative plant queries were evaluated to present a quality assessment of the presented query suggestion software.

## Design and implementation

### Distributed word vectors for a corpus based query suggestion

The design goals for LAILAPS-QSM are to provide a service for query suggestion that is (i) search corpus based, (ii) open, and (iii) extensible. Search corpus based means there is no dependency to query logs, controlled vocabulary etc. that need an elaborately maintenance. Only the text corpus itself is required that is anyway immanently available to build the text index. A “open” service implies that it should be seamlessly embeddable into existing information retrieval Web frontends or other query engines. Furthermore, this implies open access, open source, open input/output formats, and that the software should run on many platforms. Extensibility implies a design that is modular and easy to make additions to the implementation or to customize the trained query suggestion model. All of these design goals are made under consideration of an efficient implementation to enable responsive query frontends.

### Word count vectors

The LAILAPS-QSM query suggestion algorithm makes use of the word distributional hypothesis whereupon words that occur in similar contexts tend to have similar semantic meanings [12], which can be expressed by word count vectors [13]. The basic idea behind training such vectors is that the meaning of a word is characterized by its context, i.e. neighbouring words. Thus, words and their contexts are considered to be positive training samples. Because of the large data volume in life sciences, the vocabulary size and number of documents result in very large binary word distribution matrices W of dimension m × n, whereas m is the number of unique words and n is the number of documents. Taking the PubMed abstracts from last 10 years as example, m is 1.3 * 10^9^ and n is about 8.3 * 10^6^.

### Distributed word vectors

To find concepts that use distributed word vectors but meet the requirements of low memory and CPU consumption, we used the neural network language model (NNLM) [14]. A feedforward neural network with a linear projection layer and a non-linear hidden layer was used to jointly learn the word vector representation and a statistical language model. But the runtime complexity of such networks for large text corpora is still too high. Mikolov [11] published the Word2vec algorithm. It uses a log-linear neuronal network to quickly train vector representations of words from large text corpora. As illustrated by the example in Table 1, the idea is to compute for each word of a given vocabulary its influence for all possible semantic relationship that are expressed in a given text corpus. The number of expected semantic relationships is the dimension of the word vectors and need to be estimated beforehand.

**Table 1:** Example for word vector representation computed by a feedforward neural network. The word vector representations estimate the influence of a word in the context of the semantic relationship expressed by the particular word vector. This matrix is a 2 × 4 matrix, representing a vocabulary size of 4 and vector dimensions (number of expected relationships) of 2. The word vector w_1_ could represent the relationships “yield” and w_2_ “lipid source” respectively.

word	${\vec{w}}_{1}$ (“yield”)	${\vec{w}}_{2}$(“lipid source”)
produce	0.83	0.52
yield	0.99	0.34
vegetable	0.62	0.86
oil	0.41	0.92

The first suggested neural network architecture computes these word vectors as the bag-of-words model (CBOW). Here the order of words in the history does not influence the word vector. In other words, the CBOW model predicts the current word based on the context. The second neural network architecture, the Skip-gram model, tries to maximize classification of a word based on a different word in the same sentence. More precisely, this model uses the current word as an input and predict words within a certain range before and after the current word.

In both the CBOW and the Skip-gram models, the hierarchical softmax [15] function was used as an activation function in the output layer. In the traditional softmax function, we have to compute the conditional probabilities of all vocabulary words w in the context of words history, the time complexity of this computation is O(|V|). In the hierarchical softmax function, the vocabulary is represented as a Huffman binary tree based on word frequencies and the time complexity is O(log_2_|V|).

### Implementation

The implementation consists of three parts, (i) the preparation of the text corpora, (ii) the training of the query suggestion model, and (iii) the query suggestion service. To ensure platform independence, enable a high reusability and profit from year-long in-house experiences JAVA has been chosen for the implementation. To ensure a platform independent access of the query suggestion service, it was implemented as a RESTful web service.

### Text corpus building

In order to provide a ready trained web service, we trained a general purpose model with 13,930,050 documents (downloaded in June 2017) from PubMed articles, and gene and protein function descriptions from UniProt, Plant Ontology and Gene Ontology. Tokens are significant words or other units in a document. Usually whitespace characters are used as delimiters between two words. We used Apache Lucene a common text indexing system, split words based on rules from the Unicode Text Segmentation algorithm [16] and applied database name pattern from [17] to avoid the splitting of database-identifiers word pairs.

After analyzing query logs of the LAILAPS search engine [18] and The Arabidopsis Information Resource (TAIR) [19], we have found that more than 61.5% queries are composed by more than one term. The property of phrases is that their semantic meaning is beyond single word’s meanings. Therefore, the assumption is that phrases are formed by words that appear frequently together. For example, “heading date”, “salt stress” etc.

To improve the specificity of the text corpus, we add such frequent word pairs as phrases to the text corpus. To do so, we followed the approach proposed in the Word2vec algorithm and compute the correlation coefficient for counts of single words ω_i_, ω_j_ and counts of word pairs as

$$score\left(\omega_{i},\omega_{j}\right)=\frac{count\left(\omega_{i}\omega_{j}\right)-\delta }{count\left(\omega_{i}\right)*\;count\left(\omega_{i}\right)}$$

The parameter δ is used as a discounting coefficient and prevents phrases composed of very infrequent words to be formed. A suitable value for δ is determined by testing values between 1 and 20 and compare the resulting sets of extracted phrases with these later used in the results section for benchmarking the query suggestion quality. The phrases extracted for δ > 5 do not miss test set queries and a δ < 3 results in a high number but less specific phrases. As a compromise between phrase specificity and corpus size we use a δ = 5.

To get a reasonable threshold estimation for score, we computed the phrase overlap to Gene Ontology (GO), the UniProt taxonomy section, Chemical Entities of Biological Interest (ChEBI), and the Trait Ontology (TO). Iteratively, we computed the maximum overlap with ontology terms which was achieved by δ = 5 and score = 9*10^−8^. The resulting corpus size is 3,402,729 tokens including 1,465,152 phrases, whereas 20,200 phrases match to terms in the mentioned ontologies.

### Model training

Tools for text corpus tokenization, phrase extraction, and the training of the Word2Vec model [20] are bundled into one JAVA package. Beside the source code, an already compiled version is available as JAR package for an instant use in command line:

https://bitbucket.org/ipk_bit_team/bioescorte-suggestion

The final trained model is stored as a binary encoded associative list of word to word vector pairs. The size of the general purpose model [21] is 2.35GB and is loaded by the query suggestion module, usually from locally mounted storage.

### The RESTful web service

To provide the query suggestion module as a remote API to the community, a RESTful web service was implemented. It features to send a user typed query and retrieve a JSON based document with the top five alternative query suggestions. The web service documentation is available at:

 http://lailaps.ipk-gatersleben.de/client/suggestionapi.html

The endpoint URL for IPK hosted RESTful service that apply the general purpose pre-trained model is:

http://webapps.ipk-gatersleben.de/lailapssuggestion/api/v1/semanticsuggestion/<query>

Furthermore, on-site installations are possible too, by downloading a ready build web archive (WAR) file [21] and deploy it to an in-house servlet container, i.e. Apache Tomcat or Jetty. The RESTful services implement the HTTP GET verb that enables a seamless integration in any programming or script language. The URL parameter “<query>” is the original query for which you want to get suggestions. The result is returned in JSON format:

{

      “suggestions”:

      [

      <suggestions>

      ]

}

An example API call for alternatives for “heading date” is

http://webapps.ipk-gatersleben.de/lailapssuggestion/api/v1/semanticsuggestion/heading%20date

The JSON result is

{

      “suggestions”:

      [

      ”ghd7”,

      ”panicle”,

      ”tillering”,

      ”hd3a”,

      ”qtls controlling”

      ]

}

## Results

The benefit of alternative queries is to increase the search sensitivity while keeping the precision high. Using LAILAPS-QSM, we go beyond syntactic alteration or query log mining and substantially increase the potential relevant search results. For example the query “salt stress”results in 4,677 PubMed abstracts. The LAILAPS-QSM suggested, semantic similar query “salinity stress” results in 1,363 abstracts. Both have 474 (8%) abstracts in common. This is clearly a sensitivity increase by replacing one query term but staying close to the original semantic meaning.

Another benefit of LAILAPS-QSM is to apply an unsupervised machine learning to support specific training with the best fitting text corpus. This enables a tailored system that considers the individual context of the queried database. The used algorithms require less computational resources but provide a high computational performance and accuracy of the suggested queries.

A pre-trained RESTful service was deployed that was trained with PubMed, Swiss-Prot, Gene Ontology, Pfam, Plant Ontology, and Trait Ontology. This corpus comprises 8,924,354 documents and 1,434,744,725 words. It was parametrized with word vector dimensionality of 200 and applied the Skip-gram architecture. The used training dataset was retrieved from six databases:

- PubMed (query: “2007 [Date–Create]: 2017 [Date–Create]” using https://www.ncbi.nlm.nih.gov/pubmed)
- SwissProt (download SwissProt database at http://www.uniprot.org/)
- Gene Ontology Consortium (download OBO formatted data using http://www.geneontology.org/)
- Pfam (http://pfam.xfam.org/)
- Plant Ontology (http://www.obofoundry.org/ontology/po.html)
- Trait Ontology (http://archive.gramene.org/plant_ontology/ontology_browse.html).

### Evaluation of semantic relationship

The very essential prerequisite is to suggest only semantically closely related queries. Therefore, we evaluate the semantic similarity of the suggested queries to the original one. Doing so, our approach was to use plant science domain knowledge that was curated and published in ontologies. We follow the approach of Resnik’s [22] to compute the information content of the shared parent of two terms T_1_ and T_2_ as

$$Sim_{Resnik}\left(T_{1},T_{2}\right)=\text{max}(-\text{ln}\left(P\left(T_{s}\right)\right),$$

where T_s_ is the set of terms that subsume T_1_ and T_2_. P(T_s_) is the relative frequency of T_s_ in the text corpus. In other words, the similarity rate metric is the minimal relative frequency over all ontology terms that subsume both terms. A low relative frequency over a give text corpus means a special, not that common term. In contrast, a term with high frequency is commonly found in the text corpus and is used to express a broad semantic concept. If both, the original and suggested term, are successor of such a common term, they are allowed to express semantically quite different concepts. For example, if the only common parent term for “Triticum” and “Zea” would be “cellular organism” they need not to have that high semantic similarity. Because, “cellular organism” is found frequently in the training text corpus (1,456,281 times) and describes a general taxonomic classification. In contrast, if the commonly shared parent term would be “Poaceae”, both must be more similar to each other, because “Poaceae” is less commonly found (8,131 times) and describes a plant family.

This metric is efficiently to compute and has a correlation of 0.79 to human judgment. Whereas past study [23] has shown that human judgment of semantic term similarity has a correlation of 0.90. It is observed that Resnik’s metric is a compromise of reasonable high accuracy [24].

Because the value of Sim_Resnik_ is less than the information content of T_1_ or T_1_, a uniform semantic similarity (in a 0–1 scale) can be computed that follows the approach of Lin [25] as

$$Sim_{Lin}\left(T_{1},T_{2}\right)=\frac{2*Sim_{Resnik}\left(T_{1},T_{2}\right)}{-\text{ln}\left(P\left(T_{1}\right)*P\left(T_{2}\right)\right)}$$

### Evaluation results

For the evaluation, we selected 15 queries that were selected based on TAIR query logs. We requested a snapshot of the log files and got an anonymized excerpt of 571,113 queries between January 2012 and April 2013 [26]. As shown in Table 2, the queries were classified into trait, biological entity, taxonomy, and metabolic function.

**Table 2:** Test set for query suggestion benchmarking. Based on query log analysis five major classes of query can be identified: Trait, biological entity, taxonomy metabolic function. In the second column, related to each class most frequent query objectives where randomly selected from query log.

query class	subquery class	query
trait	stress response	salt stress
		drought tolerance
	agronomic traits	grain yield
	phenotypic traits	male sterility
biological entity	protein name/id	alcohol dehydrogenase
	substance name/id	dextrins
taxonomy	species name	wheat
	subspecies name	zea mays
	cultivar name	oryza glaberrima
metabolic function	catalytic process	sucrose synthase
	transport process	sucrose transporter
	primary metabolism	photosynthesis
	secondary metabolism	terpene synthase
	metabolic diseases	leaf rust
	metabolic engineering	acetolactate synthase

To compute for each query the relative frequency in the SimLin matrix, we counted the individual token frequency in PubMed abstracts. To get the common subsuming term, we used the Gene Ontology (GO) [27], the UniProt taxonomy section [28], Chemical Entities of Biological Interest (ChEBI) [29] and the Trait Ontology (TO) [30].

All 15 queries were used as input to the LAILAPS-QSM RESTful web service. For each query, we can get top 10 suggested queries, then we will try to match these suggested queries in domain ontologies, the matched results will be selected to compute the Sim_Lin_ metric. The minimal common ancestor phrases were retrieved by i) manual selection of the most similar concept for the original and suggested query and ii) the direct parent node for them. The evaluation results are compiled in S1 File. The average similarity is 0.70, whereas 34% have a score above 0.80. The minimal similarity score is 0.16, which is an outlier because “medicago sativa” is in fact not an alternative query for “zea mays”. In fact, it was suggested because some PubMed abstracts express a relationship of a different type.

In comparison to the beforehand discussed value of 0.90 for human similarity judgment, the evaluation results let us conclude that LAILAPS-QSM enhances the quality and sensitivity of database searches, particularly more relevant documents can be retrieved. The clear benefit is the capability to infer semantic similar terms directly from the source. This ensures the inclusion of recent discoveries of facts and their relation to existing knowledge. This goes beyond syntactic or rule based approaches and enables researcher to keep track with stored knowledge and avoid to miss data because of less awareness of alternative relationships to further facts.

## Availability and future directions

In this software paper, we presented the LAILAPS-QSM query suggestion RESTful web service that has been implemented under consideration of best flexibility, reusability and efficiency. Its capabilities to suggest semantically related terms to improve the sensitivity of text queries. In comparison to thesauri or query log based query suggestion implementations, the applied machine learning approach suggests even indirect existing nouns. This performs especially well for trait queries. A nice example to illustrate its useful suggestions are trait query “heading date”. Its query intention is to retrieve data that has content about the average date by which a certain percentage of a crop has formed seed heads. It is an important trait in plant breeding. The suggestions include “tillering”, “panicle”, “ghd7”, and “hd3a”. The first two suggestions are relevant traits, the latter two suggestions are genes which play an important role in rice heading date.

The provided RESTful web service in conjunction with the tool set to train based of customized text corpi enables its seamless integration into any data domain or query frontend. The memory and CPU efficient implementation supports its installation and scalable operation even using standard desktop computers. Thus, it is possible to run this service on the basis of billion words even using low-cost hardware. LAILAPS-QSM service has been already successfully included into the LAILAPS search engine [18] (see S1 Fig).

Future directions and extensions of the LAILAPS-QSM may enrich suggested query term by a semantic annotation. For example, the type labelling of an association that is expressed by the query terms would support the user to select the semantic most suited query suggestion.

## Supporting information

S1 Fig LAILAPS-QSM semantic query suggestion panel. The screenshot shows the result page for the phrase query “heading date”. The top panel displays 5 most semantically similar query terms and phrases respectively.
(TIF)

S1 File Evaluation of query suggestion similarity. This PDF file comprises the evaluation details in a tabular format. It comprises the original query, the top 5 suggested queries, and the common subsumer in the particular ontology. The Sim_Lin_ metric was computed based on the latest versions of the referenced ontologies and is shown in the correspondingly labelled column. The similarity of 1.0 represents these cases, if the suggested query is recorded as a direct synonym for the original query in the particular ontology.
(PDF)

## References

[1] Galperin MY, Fernández-Suárez XM, Rigden DJ. The 24th annual Nucleic Acids Research database issue: a look back and upcoming changes. Nucleic Acids Research. 2017;45(D1):D1–D11. doi: 10.1093/nar/gkw1188 28053160 10.1093/nar/gkw1188 PMC5210597

[2] Hersh W. Information retrieval: a health and biomedical perspective. Springer Science & Business Media; 2008.

[3] Lu Z. PubMed and beyond: a survey of web tools for searching biomedical literature. Database: the journal of biological databases and curation. 2011;2011:baq036+. doi: 10.1093/database/baq036 21245076 10.1093/database/baq036 PMC3025693

[4] Esch M, Chen J, Weise S, Hassani-Pak K, Scholz U, Lange M. A Query Suggestion Workflow for Life Science IR-Systems. Journal of Integrative Bioinformatics. 2014;11. 10.2390/biecoll-jib-2014-237 24953306

[5] Lange M, Henkel R, Müller W, Waltemath D, Weise S. Information Retrieval in Life Sciences: A Programmatic Survey In: Chen M, Hofestädt R, editors. Approaches in Integrative Bioinformatics. Berlin: Springer-Verlag; 2014 p. 73–109.

[6] Carpineto C, Romano G. A Survey of Automatic Query Expansion in Information Retrieval. ACM Comput Surv. 2012;44(1). doi: 10.1145/2071389.2071390

[7] Plansangket S. New Weighting Schemes for Document Ranking and Ranked Query Suggestion. University of Essex; 2017.

[8] Tuan LA, Kim Jj. Automatic Suggestion for PubMed Query Reformulation. Journal of Computing Science and Engineering. 2012;6(2):161–167.

[9] Doms A, Schroeder M. GoPubMed: exploring PubMed with the gene ontology. Nucleic Acids Research. 2005;33(suppl 2):W783–W786. 15980585 10.1093/nar/gki470 PMC1160231

[10] Mihalcea R, Corley C, Strapparava C. Corpus-based and knowledge-based measures of text semantic similarity. In: AAAI. vol. 6; 2006. p. 775–780.

[11] Mikolov T, Chen K, Corrado G, Dean J. Efficient estimation of word representations in vector space. ICLR Workshop. 2013.

[12] Harris ZS. Distributional structure. Word. 1954;10:146–162.

[13] Firth JR. A Synopsis of Linguistic Theory, 1930–1955 Studies in Linguistic Analysis (1957). 1957;pp:1–32.

[14] Bengio Y, Ducharme R, Vincent P, Jauvin C. A neural probabilistic language model. Journal of machine learning research. 2003;3(Feb):1137–1155.

[15] Morin F, Bengio Y. Hierarchical probabilistic neural network language model. In: AISTATS’05; 2005. p. 246–252.

[16] Davis M, Iancu L. Unicode text segmentation. Unicode Standard Annex. 2012;29.

[17] Juty N, Le Nove`re N, Laibe C. Identifiers. org and MIRIAM Registry: community resources to provide persistent identification. Nucleic Acids Research. 2011;40(D1):D580–D586. 22140103 10.1093/nar/gkr1097 PMC3245029

[18] Esch M, Chen J, Colmsee C, Klapperstück M, Grafahrend-Belau E, Scholz U, et al LAILAPS: The Plant Science Search Engine. Plant and Cell Physiology. 2015;56(1):e8 doi: 10.1093/pcp/pcu185 25480116 10.1093/pcp/pcu185 PMC4301746

[19] Lamesch P, Dreher K, Swarbreck D, Sasidharan R, Reiser L, Huala E. Using The Arabidopsis Information Resource (TAIR) to Find Information About Arabidopsis Genes In: Baxevanis AD, editor. Current Protocols in Bioinformatics. Oxford: John Wiley & Sons, Inc; 2002. 10.1002/0471250953.bi0111s30 20521243

[20] Tomas Mikolov GC Kai Chen, Dean J. Tools for computing distributed representation of words; 2015. https://github.com/tmikolov/word2vec.

[21] Chen J. RESTful API and trained model to implement a neuro-linguistic algorithms for semantic query suggestion; 2016. Available from: https://doi.org/10.5447/IPK/2016/2.

[22] Resnik P, et al Semantic similarity in a taxonomy: An information-based measure and its application to problems of ambiguity in natural language. Journal of Artificial Intelligence Research (JAIR). 1999;11:95–130.

[23] Miller GA, Charles WG. Contextual correlates of semantic similarity. Language and cognitive processes. 1991;6(1):1–28.

[24] Slimani T. Article: Description and evaluation of semantic similarity measures approaches. International Journal of Computer Applications. 2013;80(10):25–33.

[25] An information-theoretic definition of similarity. Morgan Kaufmann, San Francisco, CA; 1998.

[26] Chen J, Esch M, Lange M. Spelling correction of TAIR queries using query user refinement which has been recorded in query logs; 2014. Available from: https://doi.org/10.5447/IPK/2014/1.

[27] Ashburner M, Ball CA, Blake JA, Botstein D, Butler H, Cherry JM, et al Gene Ontology: tool for the unification of biology. Nature Genetics. 2000;25(1):25 doi: 10.1038/75556 10802651 10.1038/75556 PMC3037419

[28] Consortium U, et al UniProt: a hub for protein information. Nucleic Acids Research. 2014; p. gku989. 10.1093/nar/gku989 PMC4384041 25348405

[29] Degtyarenko K, De Matos P, Ennis M, Hastings J, Zbinden M, McNaught A, et al ChEBI: a database and ontology for chemical entities of biological interest. Nucleic Acids Research. 2007;36(suppl 1):D344–D350. 17932057 10.1093/nar/gkm791 PMC2238832

[30] Consortium PO, et al The Plant Ontology™ consortium and plant ontologies. International Journal of Genomics. 2002;3(2):137–142.

