# Supplementary figures and images for: LAILAPS-QSM: A RESTful API and JAVA library for semantic query suggestions
Source: PLoS Comput Biol. 2018 Mar 12;14(3):e1006058. doi: 10.1371/journal.pcbi.1006058 (PMC5871001; doi:10.1371/journal.pcbi.1006058)

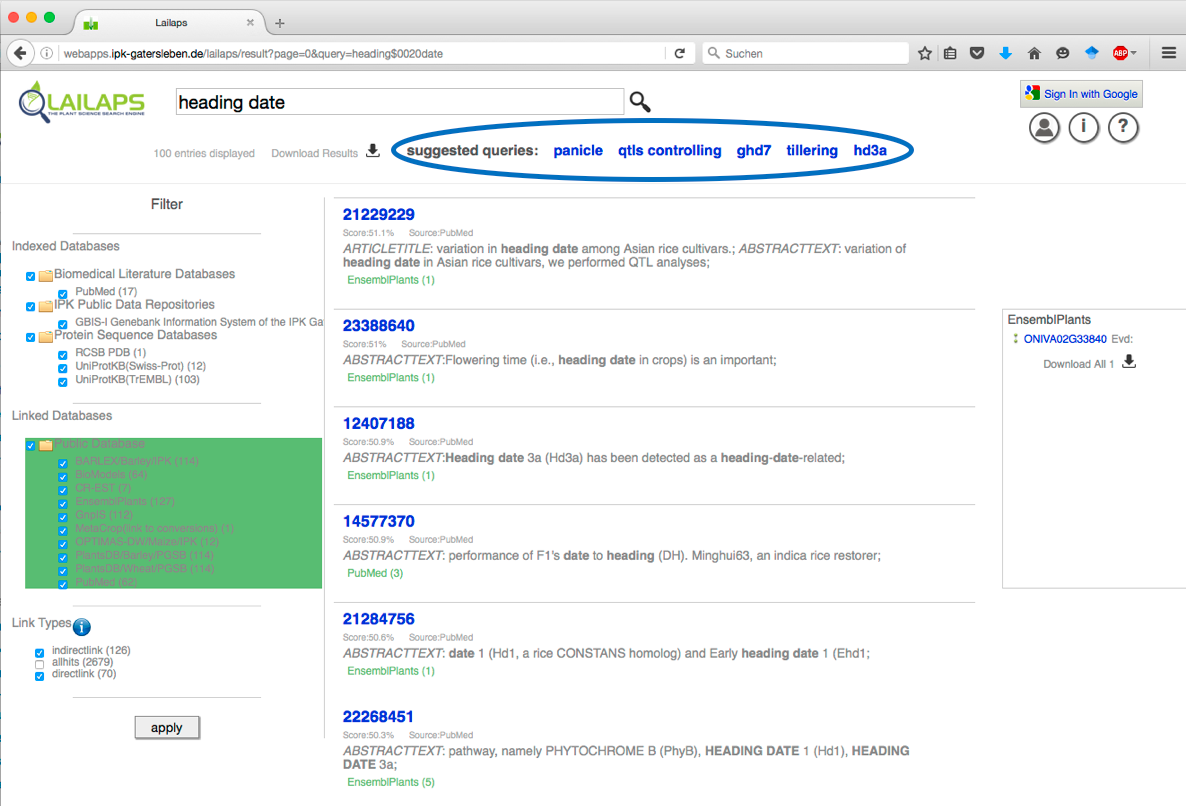

Supplement: S1 Fig — The screenshot shows the result page for the phrase query “heading date”. The top panel displays 5 most semantically similar query terms and phrases respectively. (TIF) [file pcbi.1006058.s001.tif]
