# Supplementary material for: LAILAPS-QSM: A RESTful API and JAVA library for semantic query suggestions
Source: PLoS Comput Biol. 2018 Mar 12;14(3):e1006058. doi: 10.1371/journal.pcbi.1006058 (PMC5871001; doi:10.1371/journal.pcbi.1006058)
Supplement: S1 File — This PDF file comprises the evaluation details in a tabular format. It comprises the original query, the top 5 suggested queries, and the common subsumer in the particular ontology. The SimLin metric was computed based on the latest versions of the referenced ontologies and is shown in the correspondingly labelled column. The similarity of 1.0 represents these cases, if the suggested query is recorded as a direct synonym for the original query in the particular ontology. (PDF) [file pcbi.1006058.s002.pdf]

original query	suggested query	ontology to compute Sim_{Lin}	common subsumer	Sim_{Lin} similarity
salt stress	salinity stress	Gene Ontology	response to osmotic stress	0.97
	drought stress		response to stress	0.61
	abiotic stress		response to stimulus	0.79
	chilling stress		response to stress	0.61
	drought salt		response to osmotic stress	1.00
drought tolerance	chilling tolerance	Gene Ontology	response to stress	0.53
	salt tolerance		response to stress	0.58
	salinity tolerance		response to stress	0.55
	submergence tolerance		response to abiotic stress	0.84
	cold tolerance		response to stress	0.56
grain yield	seed yield	Trait Ontology	yield trait	0.67
	tuber yield		plant trait	0.58
	panicle grain		yield trait	0.59
	tillering		yield trait	0.66
	tiller number		yield trait	0.64
male sterility	pollen sterility	Trait Ontology	sterility related trait	0.84
	sterility		plant trait	0.65
	female sterility		sterility related trait	0.85
	sterility cms		sterility related trait	0.85
	pollen fertility		sterility related trait	0.86
alcohol dehydrogenase	aldehyde dehydrogenase	Gene Ontology	oxidoreductase activity	0.89
	mannitol dehydrogenase		oxidoreductase activity	0.81
	dehydrogenase adh		oxidoreductase activity	1.0
	1.2.1.3		oxidoreductase activity	0.89
	xanthine dehydrogenase		oxidoreductase activity	0.81
dextrins	limit dextrins	Chemical Entities of Biological Interest	polysaccharide	0.68
	maltooligosaccharides		chemical entity	0.55
	arabinoxylan		polysaccharide	0.74
	amylopectin		molecular entity	0.53
	arabinoxylans		polysaccharide	0.71
wheat	triticum aestivum	UniProt Taxonomy	triticum	1.00
	maize		poaceae	0.41
	aestivum		triticum	1.00
	rye		triticeae	0.62
	soybean		mesangiospermae	0.16
Zea mays	maize	UniProt Taxonomy	zea	1.00
	aestivum wheat		poaceae	0.39
	cereale rye		triticum	0.58
	medicago sativa		mesangiospermae	0.16
	sativa rice		poaceae	0.40
Oryza glaberrima	glaberrima african	UniProt Taxonomy	oryza	1.00
	glaberrima steud		oryza	1.00
	oryza barthii		oryza	0.72
	sativa indica		oryza	0.72
	sativa ssp		mesangiospermae	0.15
sucrose synthesis	starch sucrose	Gene Ontology	cellular carbohydrate metabolic process	0.74
	sucrose starch		cellular carbohydrate metabolic process	0.74
	sucrose hexose		sucrose metabolic process	0.65
	galactolipid synthesis		cellular biosynthetic process	0.70
	enzymes calvin		carbohydrate biosynthetic process	0.80
sucrose transporter	monosaccharide transporter	Gene Ontology	carbohydrate transport	0.82
	sucrose transporters		disaccharide transport	1.00
	monosaccharide transporters		carbohydrate transport	0.83
photosynthesis	photorespiration	Gene Ontology	cellular metabolic process	0.66
	stomatal conductance		cellular process	0.68
	photosynthetic co2		cellular metabolic process	0.62
	co2 fixation		metabolic process	0.66
	acclimation photosynthetic		biological process	0.60
terpene synthesis	indole terpenoid	Gene Ontology	organic substance metabolic process	0.47
	galactolipid synthesis		lipid biosynthetic process	0.99
	mevalonate methylerythritol		isoprenoid biosynthetic process	0.88
	glucosinolate biosynthetic		cellular biosynthetic process	0.47
	triterpene biosynthetic		isoprenoid biosynthetic process	0.89
leaf rust	stripe rust	Trait Ontology	fungal disease resistance	0.71
	stem rust		fungal disease resistance	0.70
	rust resistance		fungal disease resistance	0.71
	rust wheat		fungal disease resistance	0.65
	rust stripe		fungal disease resistance	0.65
acetolactate synthase	isochorismate synthase	Gene Ontology	catalytic activity	0.60
	anthranilate synthase		catalytic activity	0.63
	linalool synthase		catalytic activity	0.58
	galactinol synthase		transferase activity	0.67
	malate synthase		transferase activity	0.70
